# Supplementary material for: Paternal-age-related de novo mutations and risk for five disorders
Source: Nat Commun. 2019 Jul 10;10:3043. doi: 10.1038/s41467-019-11039-6 (PMC6620346; doi:10.1038/s41467-019-11039-6)
Supplement: Supplementary file 1 — Supplementary Information [file 41467_2019_11039_MOESM1_ESM.pdf]

SUPPLEMENTARY INFORMATION FOR:

Paternal-age-related de novo mutations and risk for five disorders

Jacob L. Taylor[1-3], Jean-Christophe P.G. Debost[4-6], Sarah U. Morton[7], Emilie M. Wigdor[2,3,8], Henrike O. Heyne[2,3,8], Dennis Lal[2,8-10], Daniel P. Howrigan[2,8], Alex Bloemendal[2,3,8], Janne T. Larsen[4,5], Jack A. Kosmicki[2,3,8,11], Daniel J. Weiner[2,3,8], Jason Homsy[12], Jonathan G. Seidman[12], Christine E. Seidman[12-14], Esben Agerbo[4,5,15], John J. McGrath[4,16,17], Preben Bo Mortensen[4,5,15,18], Liselotte Petersen[4,5], Mark J. Daly[2,3,8], Elise B. Robinson[2,3,8,19]

[1]Department of Psychiatry, Brigham and Woman's Hospital, Boston, Massachusetts, USA. [2]Stanley Center for Psychiatric Research, Broad Institute of MIT and Harvard, Cambridge, Massachusetts, USA. [3]Program in Medical and Population Genetics, Broad Institute of MIT and Harvard, Cambridge, Massachusetts, USA. [4]National Centre for Register-based Research, Department of Economics and Business, Aarhus University, Aarhus, Denmark. [5]The Lundbeck Foundation Initiative for Integrative Psychiatric Research, iPSYCH, Aarhus, Denmark.[6]Aarhus University Hospital, Risskov, Department P, Aarhus, Denmark. [7]Division of Newborn Medicine, Boston Children's Hospital and Harvard Medical School, Boston, Massachusetts, USA. [8]Analytic and Translational Genetics Unit, Department of Medicine, Massachusetts General Hospital and Harvard Medical School, Boston, Massachusetts, USA. [9]Cologne Center for Genomics, University of Cologne, Cologne, Germany. [10]Psychiatric and Neurodevelopmental Genetics Unit, Department of Psychiatry, Massachusetts General Hospital, Boston, USA. [11]Program in Genetics and Genomics, Biological and Biomedical Sciences, Harvard Medical School, Boston, Massachusetts, USA. [12]Department of Genetics, Harvard Medical School, Boston, Massachusetts, USA. [13]Cardiovascular Division, Brigham and Women's Hospital, Boston, Massachusetts, USA. [14]Howard Hughes Medical Institute, Harvard Medical School, Boston, Massachusetts, USA. [15]Centre for Integrated Register-based Research, CIRRAU, Aarhus University, Aarhus, Denmark. [16]Queensland Brain Institute, The University of Queensland, Brisbane, Australia. [17]Queensland Centre for Mental Health Research, The Park Centre for Mental Health, Richlands, Australia. [18]Department of Biomedicine and iSEQ, Centre for Integrative Sequencing, Aarhus University, Denmark. [19]Department of Epidemiology, Harvard T.H. Chan School of Public Health, Boston, Massachusetts, USA.

Corresponding Author:
Elise B. Robinson
erobinso@hsph.harvard.edu
617-714-8541

SUPPLEMENTARY INFORMATION TABLE OF CONTENTS:

Supplementary Note 1: Proof that model accounts for different average effect sizes across subclasses of dnSNVs	*pages 3-6*
Supplementary Note 2: Estimating the impact of paternal age on expected number of dnSNVs	*page 7*
Supplementary Note 3: The model is robust to plausible levels of post-zygotic Mutations	*pages 7-9*
Supplementary Note 4: The model is robust to plausible variation in average effect size of dnSNVs	*pages 9-11*
Supplementary Note 5: Estimating the paternal age effect on ASD risk as mediated by exonic dnSNVs plus genotyped *de novo* copy number variants (dnCNVs)	*pages 11-12*
Supplementary Note 6: There is no evidence for interactions among *de novo* variants; each dnSNV exerts its influence independently	*pages 12-13*
Supplementary Note 7: Implications of the "Selfish Spermatogonial Selection" theory	*pages 13-16*
Supplementary Note 8: Alternative analyses in which we do not adjust for rate of synonymous variation	*pages 16-18*
Supplementary Note 9: Alternative analyses in which we assume that dnSNVs do not start accumulating in spermatogonial cells until age 13	*pages 18-20*
Supplementary Note 10: Why the model treats dnSNVs as accumulating proportionally with increased paternal age	*pages 20-21*
Supplementary Note 11: Discussion of maternal age effect	*pages 21-23*
Supplementary Note 12: Are increased *de novo* variants subsequent to Increasing paternal age, a likely explanation for the diagnostic expansion of autism in the United States?	*pages 23-24*
Supplementary Note 13: Determining the dnSNV rate among EPI probands	*page 24-25*
Supplementary Note 14: Determining the dnSNV rate among ASD probands	*page 25*
Supplementary Note 15: Determining the dnSNV rate among ID probands	*pages 25-26*
Supplementary Note 16: An example calculation using the dnSNV-only model	*page 26*
Supplementary Note 17: Derivation of the dnSNV-only model	*pages 26-29*
Supplementary Note 18: Comment on adjustments for maternal age and calendar time in Danish data	*pages 29*
Supplementary Note 19: An alternative approach for defining ASD in the registry data	*pages 30-32*
Supplementary Note 20: Aligning phenotypes for CHD	*pages 32-34*
Supplementary Figures 1-5	*pages 35-39*
Supplementary Tables 1-8	*pages 40-50*
Supplementary References	*pages 51-52*

Supplementary Note 1: Proof that model accounts for different effects across subclasses of dnSNVs

One important feature of the model is that it does not depend on having complete data regarding the disparate impact of different classes of *de novo* variants (e.g. missense versus protein-truncating variants) on risk. Across each disorder studied, *de novo* PTVs confer more risk compared with missense variants.[1-16] One could calculate the impact of paternal age on risk subsequent to *de novo* PTVs and missense variants separately and then determine total risk by taking the product of the associated ORs. However, the model gives the same result if one counts all nonsynonymous dnSNVs together and uses an OR reflecting the average risk conferred across all dnSNVs. This is important because while we are able to distinguish PTVs from missense variants, it is likely that for each disorder, there are many classes of dnSNVs that we cannot identify but which have distinct impacts on risk.

In this section we show that the fact that we treat all nonsynonymous variants collectively does not bias our results, even when different classes of nonsynonymous variants (e.g. missense variants versus PTVs versus PTVs within highly conserved genes, etc.) are likely to have very different impacts on disease risk:

-Assume there are g categories of dnSNVs, each of which has a distinct average effect size on disease risk. For example, two such groups might be missense variants and PTVs each of which would be expected to have different average impacts on most disease phenotypes.

-Let $X_1, X_2, \ldots, X_g$ be the average effect sizes (ORs) for each group of dnSNVs

-Let N_{01}, N_{02}, . . ., N_{0g} be the expected number of dnSNVs in each category for offspring of fathers aged 0.

-$N_0 = \sum N_{0i}$ is the expected number of total dnSNVs in offspring of fathers aged 0.

-Let N_{j1}, N_{j2}, . . ., N_{jg} be the expected number of dnSNVs in each group for offspring of fathers aged j

-$N_j = \sum N_{ji}$ is the expected number of total dnSNVs in offspring of fathers aged j.

-Let β be the coefficient from a Poisson regression of total number of dnSNVs (N) on paternal age (A): N ~ A.

-We will assume that each class of dnSNV increases at the same rate with respect to paternal age (see Supplemental Figure 4a). Thus β is also the coefficient for the regression of number of any given class of dnSNV (N_i) on paternal age.

-Therefore, $N_j = e^{\log(N_0)+j*\beta}$ and $N_{ji} = e^{\log(N_{0i})+j*\beta}$

-Let *a* be a younger paternal age and *b* be an older paternal age

-We will define X as the (geometric) mean OR of all dnSNVs in the population, weighted by expected number of each class of dnSNV. Note that because the expected number of each class of dnSNV increases at the same rate, the expected ratio between any two classes of dnSNVs is the same in offspring of fathers of any age. Therefore, across populations with different distributions of paternal ages, this ratio is expected to remain the same:

$$X = \left(\prod X_i^{N_{0i}}\right)^{\frac{1}{N_0}} = \left(\prod X_i^{N_{ji}}\right)^{\frac{1}{N_j}}$$

If we treat all classes of dnSNVs the same, or are unable to distinguish one class from another, then the expected value of our estimate of the mean effect size across all dnSNVs will be X. For example, if we cannot distinguish missense variants from PTVs, then we will treat all nonsynonymous variants collectively. X will be the mean OR reflecting disease risk across all nonsynonymous variants and N_a and N_b will be the expected number of nonsynonymous variants in offspring of fathers aged *a* and *b* respectively.

-Thus, under these conditions, the model's estimate of the paternal age effect comparing offspring of fathers aged a and b will be:

$$M_1 = X^{N_B - N_A}$$

-However, if we have enough information to treat each class of dnSNV separately (i.e. we actually know how to tell which category a given dnSNV is in and what the OR associated with that category is) then we could run the model separately for each class of dnSNV. The total paternal age effect could then be calculated as the product of the paternal age effects mediated by each class of dnSNV:

$$M_2 = \prod X_i^{N_{Bi} - N_{Ai}}$$

If we can show that the two quantities above are identical (i.e. that $M_1 = M_2$), then it suggests that failing to account for the fact that different classes of dnSNVs have

different impacts on disease risk (even going so far as to assume the possibility that each specific potential dnSNV is its own individual class) will not bias our model's estimate of the paternal age effect.

$$M_1 = X^{N_B \text{-} N_A}$$

$$= X^{e^{\log(N_0)+B*\beta} \text{-} e^{\log(N_0)+A*\beta}}$$

$$= X^{e^{\log(N_0)} * e^{B*\beta} \text{-} e^{\log(N_0)} * e^{A*\beta}}$$

$$= X^{N_0 * (e^{B*\beta} \text{-} e^{A*\beta})}$$

$$= (\prod X_i^{N_{0i}})^{e^{B*\beta} \text{-} e^{A*\beta}}$$

$$= \prod X_i^{N_{0i} * e^{B*\beta} \text{-} N_{0i} * e^{A*\beta}}$$

$$= \prod X_i^{e^{\log(N_{0i})+B*\beta} \text{-} e^{\log(N_{oi})+A*\beta}}$$

$$= \prod X_i^{N_{Bi} \text{-} N_{Ai}}$$

$$= M_2$$

This implies that treating all nonsynonymous variants collectively is a valid means of estimating the paternal age effect as mediated by *de novo* variants. Failing to account for the fact that different classes of *de novo* variants (and different specific variants within any class) have differential impacts on each disorder does not adversely impact the validity of this model.

Supplementary Note 2: Estimating the impact of paternal age on expected number of dnSNVs

In the main text, we describe an analysis using Poisson regression to explore the relationship between number of dnSNVs in each unaffected sibling of an ASD probands (n = 1821) on paternal age at birth. We ran similar models using: 1) all exonic dnSNVs, 2) synonymous dnSNVs, 3) missense variants, and 4) protein-truncating variants (PTVs). We found no evidence that dnSNV accumulation as a function of paternal age differed across classes of variant (Supplementary Figure 2). These results are highly consistent with previous studies that used whole-genome sequencing to investigate the association between parental age and dnSNV rate (see Supplementary Table 3).

Supplementary Note 3: The model is robust to plausible levels of post-zygotic mutations

A substantial portion of the dnSNVs that have been called across various studies may have arisen shortly after the formation of a zygote rather than through the germline. A recent report estimates that 7.5% of dnSNVs may in fact be postzygotic mosaic mutations (PMMs).[17] This estimate is in-line with other estimates from smaller samples. Since PMMs would not be expected to bear any relationship with paternal age, it seems plausible that counting these mutations incorrectly as germline-derived dnSNVs could distort our results.

To explore this concern, we assumed that 7.5% of measured dnSNVs in each sample was a post-zygotic mutation (PZM) and that PZMs remain constant with respect to paternal age. Recall that we are estimating the marginal impact of dnSNVs on disease

risk by dividing the mean number of dnSNVs per person in a group of individuals with a disorder, by the mean number per person in a group of controls:

$$\mathrm{OR}_{\mathrm{dnSNV}} = {}^{\#\mathrm{dnSNV\ per\ case}}/_{\#\mathrm{dnSNV\ per\ control}}$$

There is no reason to assume that the proportion of measured dnSNVs that are actually PZMs is different in cases compared with controls. Since PZMs that have large enough allele frequencies to potentially be called as dnSNVs arise very early in development, they can also be causally related to any of the disorders considered in this paper. Thus, from the equation above, we see that assuming that a certain proportion (e.g. 7.5%) of measured dnSNVs are actually PZMs would not affect our estimate for the OR associated with a true dnSNV:

$$\mathrm{OR}_{\mathrm{dnSNV}} = {}^{0.075*\#\mathrm{dnSNV\ per\ case}}/_{0.075*\#\mathrm{dnSNV\ per\ control}} = {}^{\#\mathrm{dnSNV\ per\ case}}/_{\#\mathrm{dnSNV\ per\ control}}$$

One reasonable way of modelling the impact of mis-calling PZMs as dnSNVs on our estimate for the relationship between dnSNVs and paternal age in the general population is to assume that each individual, regardless of paternal age, has a fixed expected number of PZMs, that should be subtracted from whatever their total expected number of measured dnSNVs is. For example, let us assume that 7.5% of the 1,214 measured dnSNVs among the n=1827 SSC siblings are actually PZMs. This means that each SSC sibling has, on average, (1214*0.075)/1827 = 0.05 PZMs regardless of paternal age. We know that the total number of measured dnSNVs fits well according to the equation:

$$\text{Expected \#dnSNVs} = e^{-1.45+\text{age}*0.031}$$

Thus, we could estimate the expected number of true dnSNVs as:

$$\text{Expected \#dnSNVs} = e^{-1.45+\text{age}*0.031} - 0.05$$

However, modifying the expected number of true dnSNVs as a function of age in this way, will have no impact on our model. Our model involves exponentiating the OR associated with a single dnSNV by the difference between the number of expected dnSNVs at an older age and number of expected dnSNVs at a younger age. Since our estimate of the number of dnSNV at each age would be changed by the same constant (0.05 in this case), the difference between these two quantities will not change. Thus, even if a certain proportion of measured dnSNVs are PZMs, it will not affect the output of this model. More generally, even if we assume that a certain proportion of dnSNVs are generated by a mechanism that is not related to paternal age, as long as the proportion of such dnSNVs is the same across people with different disorders and no relevant disorder, we would not expect this phenomenon to substantially impact the estimates from this model.

Supplementary Note 4: The model is robust to plausible variation in average effect size of dnSNVs

In order to ensure that the IRRs obtained by this method are robust to our choice to use the SSC siblings to estimate the "expected" rate of dnSNVs per person, we tested two alternative methods for generating ORs reflecting ASD risk given one additional nonsynonymous dnSNV. We did these analyses using only the ASD probands for two reasons, both related to the fact that our control subjects were recruited

through the same study as the included ASD probands. First, this was the only population where we had systematic and reasonably complete data on paternal age at birth of probands with a disorder. Second, this was the only population in which we had large-scale genotyped *de novo* copy number variant (dnCNV) data. This made the ASD cohort an ideal population for evaluating how the particular choices we made in estimating the impact of *de novo* variation on risk for a disease, could impact the inferences drawn from our model.

The first alternative method for estimating the impact of dnSNVs on disease risk involved regressing number of dnSNVs within offspring (ASD cases and siblings) in the SSC trio and quad families by case/sibling status while controlling for paternal age. The exponentiated regression coefficient for case status provides an alternative estimate for the OR associated with one additional dnSNV. We also ran a model in which we additionally included number of synonymous variants. This had no effect on the estimated impact of non-synonymous variants (beta changed from 0.1707 with SE of 0.0366 to 0.1708 with same SE). Note that because neither data on paternal ages, nor a sibling control group, were available for the other disorders, this method for estimating the effect of dnSNVs could only be applied to ASD. However, because we adjusted for the rate of *de novo* synonymous variation across all disorders, any systematic differences in parental ages between the cohorts would be unlikely to distort the results.

Second, instead of using the SSC siblings as our source to estimate the baseline-rate of nonsynonymous dnSNVs, we used a model of *de novo* expectation based on Samocha et al.'s (2014) genome-wide analysis of sequence context.[18] This model

provides a very similar estimate of the number of nonsynonymous dnSNVs per person (0.71) compared to the estimate obtained by directly counting dnSNVs in the SSC siblings (0.66). Because there is no standard error associated with the estimate obtained by sequence context, it is not possible to rigorously test whether these two estimates are statistically different from one another. It is largely because we were able to quantify our uncertainty about the expected number of dnSNVs using the SSC siblings that we used this population to find the baseline rate in our main analyses. Supplementary Figure 1 illustrates that the choice of method for estimating the marginal impact of a single dnSNV in ASD makes very little difference, even in a comparison of risk in offspring of 55-year-old versus 25-year-old fathers.

Supplementary Note 5: Estimating the paternal age effect on ASD risk as mediated by exonic dnSNVs plus genotyped *de novo* copy number variants (dnCNVs)

Sanders et al. (2015)[19] provided dnCNV data from the SSC for ASD probands and their unaffected siblings. We used those data to extend our model to include dnCNVs in the 2,346 ASD probands and 1,761 unaffected siblings (along with their parents) who were: 1) whole-exome sequenced, 2) genotyped for dnCNVs, and 3) had available data on paternal age at birth. First, we regressed number of dnCNVs against paternal age in the SSC siblings. There was no statistical evidence that rate of dnCNVs was associated with paternal age (p = 0.27 against the null hypothesis of no association between rate of dnCNVs and paternal age). This null finding, however, could reflect a problem of statistical power. As only a limited number (n=45) of SSC siblings had a dnCNV, we were also unable to detect a statistical difference between the paternal age-dnCNV

association and the paternal age-dnSNVs association (p = 0.98) (see Supplementary Figure 4b), despite very strong statistical evidence for the latter.

For purposes of examining the potential influence of dnCNVs on our estimates of the paternal age effect, we made the conservative choice to treat dnSNVs and dnCNVs as if they increase with paternal age at the same rate. We then regressed total number of *de novo* variants (dnSNVs + dnCNVs) on proband/sibling status while controlling for paternal age in order to estimate the OR associated with a single *de novo* variant of either class. We were therefore able to apply the full paternal age model to estimate the paternal age effect as mediated by both dnSNVs and genotyped dnCNVs. As shown in Supplemental Figure 1, including dnCNVs in the analysis does not substantially impact our estimate for the effect of increased paternal age (offspring of 55-year-old fathers versus offspring of 25-year-old fathers) on ASD risk as mediated by *de novo* variation. Although the marginal impact of individual dnCNVs on ASD risk (OR of 2.61) is substantially higher than the marginal impact of single dnSNVs, dnCNVs are comparatively uncommon (seen in 2.6% of controls compared with nonsynonymous dnSNVs which are found in 46.2% of controls) and accordingly do not substantially add to our estimates of a paternal age effect. Across disorders, it is unlikely that additional classes of *de novo* variation are both sufficiently common and sufficiently impactful to substantially change inferences produced by this model. $^{\mathrm{A} * \mathrm{D}}/_{\mathrm{B} * \mathrm{C}}$

Supplementary Note 6: There is no evidence for interactions among *de novo* variants; each dnSNV exerts its influence independently

Our model assumes that in individuals with > 1 dnSNV, each dnSNV acts independently. We tested this assumption in the SSC families by examining the probability of being a proband vs. a sibling in individuals with one versus zero dnSNVs and exploring whether there was any statistical evidence of non-additivity in having 2 vs. 1 dnSNV, 3 vs. 2, 4 vs. 3 or 5 vs. 4 dnSNVs. We did this by taking the natural logarithm (ln) of each of these ORs and calculating the standard errors of each ln(OR) using $\sqrt{(1/_a) + (1/_b) + (1/_c) + (1/_d)}$ where a = number of cases with n dnSNVs, b = number of siblings with n dnSNVs, c = number of cases with n+1 dnSNVs and d = number of siblings with n+1 dnSNVs. We were then able to do z tests to compare the OR associated with one versus zero dnSNVs to each other OR. The resulting p values from these z tests were 0.89, 0.46, 0.88 and 0.22 respectively, suggesting no evidence for interactions among dnSNVs, at least with respect to ASD. We are not aware of any evidence across any human disorder that at a population level, *de novo* variants act non-additively in conferring risk.

Supplementary Note 7: Implications of the "Selfish Spermatogonial Selection" theory

The "Selfish Spermatogonial Selection" (SSS) theory was posited as a possible contributor to the relationship between advanced paternal age and neuropsychiatric disease.[20] It builds off findings in certain rare paternal-age associated Mendelian disorders in which the causal mutations seem to provide a within-testis selection benefit to spermatogonial cells in which they arise. The authors note that if mutations which give spermatogonial cells a selective advantage were more likely than other mutations

to increase risk for a disorder, then the impact of advanced paternal age on risk, for that disorder, would be increased beyond what would be expected if disease-related mutations accumulated through an entirely random process. They point to some evidence that rare variants implicated in ASD as well as candidate genes hypothesized to be important in SCZ, may involve molecular pathways that are also involved in spermatogonial cell proliferation.

In the model described in the present paper, there is an assumption of independence between the likelihood that a particular mutation arises as a result of advancing paternal age and the likelihood that this mutation causes disease. To the extent that this assumption is violated in the direction proposed in the SSS theory, the present model would tend to underestimate the impact of paternal age-associated dnSNVs.

There are three main reasons why the phenomenon of disorder related dnSNVs accumulating preferentially in sperm cells with advancing paternal age is unlikely to have a substantial impact on the results described in this paper. First, there is no direct empirical evidence that a substantial portion of mutations that cause any of these disorders also confer a selective advantage on spermatogonial cells. There is some evidence that SSS may account for some cases of mendelian disorders such as Apert syndrome and achondroplasia. It is plausible that SSS may lead to the accumulation of mutations in genes involved in the RAS-pathway which can be associated with neurodevelopmental disorders. However, at present there is no empirical evidence that this mechanism causes a substantial portion of any of these disorders at the population level.

Second, in their simulation study, Gratten et al [21] specifically explore whether SSS could plausibly account for a substantial portion of the paternal age effect in ASD and SCZ. They find that across all plausible combinations of parameters, SSS would be unlikely to confer more than 1% additional risk beyond baseline in offspring of fathers of advanced age.

Finally, we conducted an exploratory empirical analysis to test a major assumption of the SSS theory as applied to ASD. If a substantial portion of the increased risk of ASD with advanced paternal age were due to *de novo* mutations that give spermatogonial cells in which they arise a selective advantage, one would expect that such mutations would become an increasingly large portion of all *de novo* mutations as paternal age advances. In other words, a typical dnSNV inherited from an older father would be expected to confer greater risk for developing an ASD than a typical dnSNV inherited from a younger father.

We tested this hypothesis using a logistic regression model in the SSC trio and quad families, in which disease status is the outcome (1 = case, 0 = control) and the explanatory variables include number of dnSNVs, paternal age and an interaction term between the two. If dnSNVs acquired from older fathers were more likely to cause ASD than dnSNVs acquired from younger fathers, we would expect the interaction term to be greater than 0.

We ran this analysis in n = 4273 individuals (2446 cases) with available paternal age and dnSNV data. We found positive and statistically significant associations between case status and both number of dnSNVs (p = 0.008) and paternal age (p = 0.0002). However, contrary to expectations if the SSS model were a driver of the paternal-age

relationship, the interaction term was negative (and with a two-sided p-value of 0.16 against the null hypothesis that its true value is zero). Furthermore, when dividing the sample into subjects with paternal age greater than 34.2 (60th percentile) and those with paternal age less than 31.6 (40th percentile), the estimated impact of a single dnSNV is less in the older group (beta in logistic regression model = 0.15 (SE = 0.06) versus 0.09 (SE = 0.05)). In summary, in the SSC families, dnSNVs inherited from older fathers are not more likely to be causally related to ASD compared to dnSNVs inherited from younger fathers.

Thus, due to (1) the absence of empirical evidence for the relevance of the SSS model for any of the disorders looked at, (2) a simulation result suggesting that it is implausible that this model is a substantial driver of paternal-age associations in genetically complex disorders and (3) an empirical analysis in ASD trio and quad families which suggest a trend in the opposite direction of that predicted by this model, we believe it would not be reasonable to attempt to account for this model in the present investigation.

Supplementary Note 8: Alternative analyses in which we do not adjust for rate of synonymous variation

In our main analysis, we made the assumption that synonymous variation – mutations that do not cause changes to amino acid sequences – should occur at the same rate across cohorts. This assumption is generally accepted as valid, though it is biologically plausible that de novo synonymous variants could be slightly more common in individuals with genetically influenced disorders. Although synonymous variation does

not affect amino acid sequences, it could affect the rate at which different RNA sequences are transcribed, which could theoretically effect risk for disease. Furthermore, in disorders that are associated with parental age for reasons unrelated to de novo variation, we would expect probands to have slightly older parents, who would therefore pass on slightly more dnSNVs of all types, including synonymous variants. For these reasons, adjusting for rate of synonymous variation could subtly bias our conclusions about the marginal impact of nonsynonymous dnSNVs toward the null and could therefore cause us to slightly under-estimate the paternal age effect across disorders. In addition, because our calculation of confidence intervals (see below) involves the propagation of uncertainty across all parameters, the inclusion of additional parameters (average number of synonymous *de novo* variants within a population) increases the uncertainty around our estimates of the paternal age effect due to *de novo* variants.

Therefore, we performed alternative analyses using the unadjusted ORs (and associated standard errors) from Table 1 of the main text. Results of these analyses are summarized in Supplementary Tables 1 and 2 and Supplementary Figures 2 and 3. Across disorders, not adjusting for synonymous rate had the effect of shrinking confidence intervals around the estimates generated by our dnSNV model. This effect, plus a small decrease in our estimate of the impact of a typical dnSNV on SCZ risk, led to an increase in statistical confidence that the epidemiologic paternal age effect in Denmark cannot be accounted for solely by dnSNVs ($p = 10^{-4}$). An increased estimate of the impact of a typical dnSNV on risk for ID, led to the disappearance of a marginally-

apparent difference between the epidemiologic and dnSNV models (p = 0.24, up from p-value of 0.05 when using the dnSNV model that adjusts for synonymous rate).

Supplementary Note 9: Alternative analyses in which we assume that dnSNVs do not start accumulating in spermatogonial cells until age 13

The model described in the main text assumes that dnSNVs accumulate at the same proportional rate beginning at birth. Biologically, it is more likely that the rate of accumulation of dnSNVs is close to zero until the onset of puberty, when spermatogonial cells begin going through regular mitotic events. To explore whether the choice described in the main text might substantively distort the results reported, we repeated the analyses described while making the assumption that the average number of dnSNVs per exome is fixed from birth through age 13 and that this number increases thereafter.

We first re-ran the Poisson regression analysis using the unaffected siblings of autism probands from the SSC data set. In the main analysis we regressed number of dnSNVs in each SSC sibling against paternal age at that individual's birth (see Supplementary Note 2). In the present analysis we regressed number of dnSNVs in each SSC sibling against paternal age at that individual's birth minus 13 years. Unsurprisingly, the coefficient (β) representing the proportionate increase in number of dnSNVs for each year of increased paternal age remains unchanged at 0.031. The standard error for the estimate of this parameter also remains unchanged. What does change, however, is the estimate of the intercept (i) for this model, which corresponds to the natural log of the estimated number of dnSNVs per exome prior to any age-related accumulation in

this number. For the analysis reported in the main text, i = -1.45, corresponding to an estimate of 0.23 ($e^{-1.45}$) dnSNVs per exome when the SSC fathers were age = 0. In the present analysis rate, i = -1.05, corresponding to an estimate of 0.35 ($e^{-1.05}$) dnSNVs per exome when the SSC fathers were age = 13 years. Most significantly for downstream modelling, because we are assuming a shorter time scale over which age-related dnSNVs could have accumulated, the standard error around the estimate for i is smaller in the present analysis (0.11 vs. 0.17).

The model described in Equation 1 (main text) remains essentially unchanged by the assumption that dnSNVs do not start accumulating until age 13. None of the estimates for the impact of age-related dnSNVs as presented in main text Figures 1 and 2, and Supplementary Table 4 are different. However, because the uncertainty around the *i* parameter is smaller, the uncertainty around the quantity "#nonsyn(age)" (see main text Equation 1) is smaller. This decreases the uncertainty in our estimates of the predicted changes in population-level disease incidence due to paternal-age related dnSNVs in the exome. Thus, making the assumption that age-related dnSNVs do not start accumulating in the exome until age 13 leads to slightly smaller confidence intervals around the results reported in this paper. This in turn leads to slightly smaller p-values when testing whether our estimates for the paternal-age related increases disorder incidence is different from the paternal-age associated differences in incidence observed in the Danish epidemiologic data (see Supplementary Table 5).

These differences are not substantial enough to change any inferences. There remains strong statistical evidence that the observed associations between paternal age and ASD is greater than what could reasonably be explained by dnSNVs (p value goes

from 2e-5 to <2e-5 for ASD), borderline evidence for SCZ/ID (p value goes from 0.02 to 0.006 for SCZ and from 0.05 to 0.02 for ID) and no statistical evidence for differences for CHD and EPI (p > 0.2 in all cases).

We choose to use the assumption that dnSNVs accumulate at a steady rate starting at age 0 in reporting the main results for three reasons. First, different individuals begin puberty at different ages. Making the assumption that puberty begins at age 13 (or any other particular age) in males across the board doesn't ultimately change the fact that there are subtle ways in which this model may not perfectly capture biological reality. Second, one of the appeals of the model as presented in the main text is its relative simplicity. Adding in another parameter (estimated age at which puberty begins) that increases the model's complexity while changing almost nothing with respect to the model's outputs seems like an unnecessary sacrifice of clarity and interpretability. Finally, the only change in the output of the model by making the assumption that dnSNVs do not start accumulating until age 13 is to increase our apparent confidence in the model's outputs. Thus, presenting the model as it is described in Equation 1 (main text), rather than as it is described in this Supplementary Note seems to be the more conservative choice.

Supplementary Note 10: Why the model treats dnSNVs as accumulating proportionally with increased paternal age

In the results section, we report that dnSNVs accumulate at a rate of 3.1% per year of increased paternal age. This comes out of the Poisson model run using data from the 1821 unaffected siblings of ASD probands from the SSC, in which the beta for paternal age was 0.031.

Because dnSNVs are, for the most part, likely to occur independently of one another, we believe it is reasonable to model their occurance using Poisson regression. Furthermore, as noted Supplementary Note 1, the choice to model dnSNVs as increasing proportionally allow us not to distinguish between different classes of dnSNVs (such as missense variants and PTVs) which have different average effect sizes on disease phenotypes.

In order to explore whether modelling the accumulation of dnSNVs with paternal age using Poisson regression is a reasonable approach to modelling the data, we divided the SSC controls into deciles according to paternal age. We compare the mean number of dnSNVs for each decile to the number predicted according to the proportional model we used in this paper. We also compared it to a linear model. The parameters for this linear model were found by linearly regressing number of dnSNVs per proband by paternal age: intercept = -0.046, Beta = 0.022 ($p = 10^{-9}$) with respect to the null hypothesis that Beta = 0. Visual inspection of resulting figures (Supplementary Figures 5a and 5b) suggest that both the proportional model and the linear model are reasonable approximations of the available data. Since the proportional model allows us to estimate the paternal age effect without detailed knowledge of the heterogeneous effect sizes of different classes of dnSNVs on each disorder, we believe it is most appropriate to use this model.

Supplementary Note 11: Discussion of maternal age effect

Paternal and maternal ages at birth were highly correlated among the SSC parents (Pearson coefficient = 0.72, 95% confidence interval 0.70 – 0.74). We determined this correlation coefficient and its confidence interval using the R function "cor.test" which generates a test statistic based on Pearson's product moment correlation coefficient. When both paternal and maternal ages were included in a

regression model predicting number of *de novo* nonsynonymous single nucleotide variants (dnSNVs) in offspring, our results suggested that we could not reliably model their independent effects given the degree of correlation. Specifically, when we regressed number of synonymous variants on maternal and paternal ages we obtained a coefficient of 0.030 (p= 0.03) for maternal age and a coefficient of -0.001 (p=0.92) for paternal age. By contrast, when we regressed number of nonsynonymous variants on maternal and paternal ages we obtained a coefficient of 0.028 ($p=5e10^{-5}$) for paternal age and a coefficient of 0.006 (p=0.46) for maternal age. This pattern of associations does not seem plausible, and we concluded that paternal and maternal ages were too highly correlated to usefully adjust for co-parent age in our models.

Published results on dnSNVs from whole-genome-sequenced trios suggest that dnSNVs of paternal origin are 3-4 times as prevalent as those of maternal origin.[24-26] Thus, even if dnSNVs accumulated at the same relative rate in male and female germ cells, advanced paternal age would be expected to have a substantially greater influence on dnSNVs in offspring compared with advanced maternal age. As there does appear to be a small true association between dnSNVs and maternal age,[14,26] it is likely that a portion of our predicted increase in dnSNVs associated with increased paternal age is attributable to the fact that children with older fathers also tend to have older mothers, and that some of the excess dnSNVs are received from these older mothers.

There are implications to our not accounting for the potential role of maternal age in our model. First, if one were to control for maternal age, then the magnitude of the paternal age related dnSNV effects would likely be slightly smaller than those reported in our study. Second, our model will be most accurate for comparing risk in

offspring of parents whose age difference is similar to typical parents in the SSC quad families. In this sample fathers were, on average, 2.2 years older than mothers (sd = 4.0 years). This average difference is approximately the same as the likely father-mother age difference in the Danish population, where men were, on average, 2.4 years older than women when first married in 2015 (34.3 - 31.9 years).[27,28]

Supplementary Note 12: Are increased *de novo* variants subsequent to increasing paternal age, a likely explanation for the diagnostic expansion of autism in the United States?

Prevalence rates of the broader autism spectrum was approximately 0.001 in population-based studies published from the 1960s-1990s.[22] In 2012, the prevalence of ASD was approximately 0.015.[23] A single dnSNV increases risk of ASD by a factor of 1.20 (Table 1). Paternal age is related to the expected number of dnSNVs according to the equation:

$$\text{\# of dnSNVs} = e^{-1.45 + 0.031*\text{Age}}$$

If we assume (conservatively) that the mean paternal age at the time that ASD prevalence was measured as 0.001was 25 and if we assume the increase in measured prevalence was due to paternal age-associated dnSNVs, then we can algebraically determine what the mean paternal age in the population must have been in 2012:

(1) First, use the relationship between previous prevalence (0.001) and assumed mean paternal age (25) to estimate "baseline prevalence" (the prevalence in individuals with zero dnSNVs):

$$0.001 = \text{Baseline}*1.20^{e\wedge(-1.45 + 0.031*25)}$$

Baseline = 0.001/1.20$^{e\wedge(-1.45 + 0.031*25)}$ = 4.2x10^{-4}

(2) Next, use the relationship between prevalence in 2012 and paternal age to solve for mean paternal age in 2012, while assuming the "baseline prevalence" has not changed:

0.015 = (4.2*10^{-4})*1. 20$^{e\wedge(-1.45 + 0.031*AGE)}$

0.015/(4.2*10^{-4})= 1. 20$^{e\wedge(-1.45 + 0.031*AGE)}$

$$\frac{\log\left({}^{0.015}/_{9.1*10^{-4}}\right)}{\log(1.22)} = e^{-1.45+0.031*AGE}$$

$$\log\left(\frac{\log\left({}^{0.015}/_{9.1*10^{-4}}\right)}{\log(1.22)}\right) = -1.45 + 0.031*AGE$$

$$AGE = \frac{\log\left(\frac{log\left({}^{0.015}/_{9.1*10^{-4}}\right)}{log(1.22)}\right) + 1.45}{0.031} = 132.1$$

Thus, we conclude that if the prevalence of ASD really did increase from 0.001 to 0.015 in recent years and this increase were driven by an increase in dnSNVs due to delayed paternity from a baseline paternal age of 25, mean paternal age at birth would now have to be 132 years old.

Supplementary Note 13: Determining the dnSNV rate among EPI probands

Because some *de novo* variants belong to two transcripts, it is sometimes ambiguous as to whether a particular variant is nonsynonymous or synonymous (no direct impact on the amino acid sequence of a protein). The data we used for estimating the dnSNV rate in ASD, SCZ, CHD and ID is based on assigning ambiguous variants to the

canonical transcript. However, the data we used for estimating the dnSNV rate in EPI was based on a choice to assign each *de novo* variant to whichever transcript would result in the most severe consequence. Relative to the other disorders, this choice would cause the dnSNV rate in EPI to appear inflated. Fortunately, the study reporting the dnSNV rate in epilepsy also re-called *de novo* variants in 1911 SSC controls using the same approach. They found a slightly higher rate of dnSNVs among controls (0.69 per person). Thus, for all analyses involving EPI, we used this slightly higher rate among controls.

Supplementary Note 14: Determining the dnSNV rate among ASD probands

For ASD, there are two non-overlapping studies in which trio families were whole-exome-sequenced. [1,6]. Only the study reporting on the SSC trio-families provided detailed enough phenotypic data (i.e. proportion of probands who meet DSM-IV criteria for ASD vs. pervasive developmental disorder vs. Asperger's Syndrome) to make comparison with the Danish registry data practical.[1] Therefore, we only included data from this study in our main analysis. However, even if we had included the other study,[6] it would not have changed any inferences made about the expected magnitude of the effect of paternal age on ASD subsequent to dnSNVs as the reported rates of nonsynonymous dnSNVs per proband were virtually identical between the two studies (0.79 in the study that we used and 0.78 in the excluded study).

Supplementary Note 15: Determining the dnSNV rate among ID probands

For ID we used the rate reported by Samocha et al (2017)[29], who calculated the rate of dnSNVs across 5264 non-overlapping trio probands, ascertained through five studies of ID[10-12] or severe developmental delay[13,14] (where at least 90% of probands have significant ID).[13] Data from 1077 non-overlapping trio probands with schizophrenia were obtained from 7 studies.[2-5,7,8,30] For EPI, data were obtained from a single study of epileptic encephalopathy and neurodevelopmental disorders with epilepsy.[15] For CHD, data were obtained from the most recent study published by the Pediatric Cardiac Genomics Consortium.[31]

Supplementary Note 16: An example calculation using the dnSNV-only model

The OR for ID associated with one additional dnSNV is 1.50. The expected number of nonsynonymous dnSNVs in offspring of 25-year-old fathers is 0.51 ($e^{-1.45 + 0.031*25}$). Thus, the expected incidence of ID in offspring of 25-year-old fathers is $INC_{baseline}*1.50^{0.51}$. Similarly, because the expected number of nonsynonymous dnSNVs in offspring of 45-year-old fathers is 0.95, the expected incidence of ID in their offspring is $INC_{baseline}*1.50^{0.95}$. Therefore, to find the IRR in offspring of 45-year-old fathers compared to offspring of 25-year-old fathers, one takes the quotient of the two quantities above:

$$INC_{baseline}*1.50^{0.95} / INC_{baseline}*1.50^{0.51}$$

This reduces to: $1.50^{0.95 - 0.51} = 1.20$.

Supplementary Note 17: Derivation of the dnSNV-only model

Here we discuss some of the underlying assumptions and the derivation of the *de novo*-only model

Assumptions:

(1) *De novo* variants occur independently from one another. Conditioned on paternal age they are Poisson distributed.

(2) The marginal impact of each dnSNV is independent of others (i.e. there are no statistical interactions among dnSNVs). So, for example, the increased risk associated with having two dnSNVs is expected to be the increased risk associated with having one dnSNV squared. Above (Supplementary Note 3) we show that there is no evidence in the ASD data for an interaction.

(3) The rate of change with respect to number of dnSNVs and paternal age is the same across classes of dnSNVs. Therefore, we assume there is no relationship between the probability that a particular dnSNV will arise with increased paternal age and the expected impact of that dnSNV on disease risk. Supplementary Figure 4a illustrates that there is no evidence in the SSC siblings that increasing paternal age differentially impacts dnSNVs of particular classes.

(4) Multiplying a baseline incident rate for a disorder in a population of people with zero dnSNVs by the odds ratio reflecting risk associated with a certain expected number of dnSNVs should give a reliable estimate of the incidence rate in the population of individuals with that number of dnSNVs. Since odds ratios are approximately equivalent to rate ratios for relatively rare disorders, this assumption is valid for the disorders considered here.

Inputs:

i = An estimate of the natural log of the expected number of dnSNVs in offspring of fathers “aged zero” in the general population. This term is the intercept from Poisson regressing number of dnSNVs in siblings of autism probands (n = 1821) against paternal age at birth of these siblings.

β = An estimate of the increase in number of dnSNVs per year of increased paternal age. This term is the coefficient from the same Poisson regression.

OR = An estimate of the odds ratio associated with the increased risk of having a disease given one additional dnSNV

a = any given paternal age

b = any given paternal age that is greater than *a*

Derivation:

(1) The expected number of dnSNVs in offspring of fathers aged a = $e^{i + a*\beta}$

(2) The expected number of dnSNVs in offspring of fathers aged b = $e^{i + b*\beta}$

(3) Following assumption (2) above, the odds ratio associated with the increased risk of having a disease given n additional dnSNVs is OR^n

(4) Therefore, compared to an individual with 0 dnSNVs, an individual with n dnSNVs has odds that are OR^n times greater of having the disease

(5) Offspring of fathers aged a will have odds that are OR ^ ($e^{i + a*\beta}$) times greater than individuals with 0 dnSNVs of having the disease

(6) Offspring of fathers aged b will have odds that are OR ^ ($e^{i + b*\beta}$) times greater than individuals with 0 dnSNVs of having the disease

(7) To determine the incident rate ratio reflecting the increased risk of disease in offspring of fathers aged b compared with offspring of fathers aged a we divide the previous two quantities: OR ^ ($e^{I + b*\beta}$) / OR ^ ($e^{I + a*\beta}$)

(8) This reduces to: OR ^ ($e^{I + b*\beta} – e^{I + a*\beta}$) which is equivalent to the expression contained in Equation 1 (main text).

Supplementary Note 18: Comment on adjustments for maternal age and calendar time in Danish data

Supplementary Table 7 shows that, overall, adjustment for maternal age and calendar time does not have a statically significant impact on estimates of the epidemiologic paternal age effect in Denmark. Furthermore, using any of the adjusted estimates of the epidemiologic association between advanced paternal age and disease risk, does not change the inferences described in the paper: that accumulating *de novo* variants could plausibly account for any paternal age effect on risk for ID, EP and CHD, while these could not fully explain the larger epidemiological associations between advanced paternal age and ASD and SCZ.

The fact that we did not see any difference by adjusting for maternal age or calendar time, could be an issue of power. As we are not powered to detect a significant change in the OR for paternal age when controlling for either of the variable of interest, we did not include a model that contains both, as this could create inflate the type 1 error rate.

Supplementary Note 19: An alternative approach for defining ASD in the registry data

For ASD, we queried the Simons Simplex Collection (SSC) database to determine the distribution of specific DSM-IV ASD clinician diagnoses. The clinician diagnoses are documented in the 'nonstandardized_impressions' variable. Nearly 70% (69.3%) of probands had a diagnosis of autistic disorder. The remainder had diagnoses of either Asperger's disorder or pervasive developmental disorder not otherwise specified (PDD-NOS). Clinicians who evaluated SSC probands at different sites often disagreed on specific ASD diagnoses suggesting that distinctions between individuals with different ASD diagnoses were not reliable.[32] Furthermore, these specific diagnoses were replaced by the global 'autism spectrum disorders' category in the DSM-V. [33] For these reasons, as well as concern that the phenotypic variance would likely be greater among Danish individuals with diagnoses of Asperger's disorder or PDD-NOS compared with autistic disorder, we limited our primary analysis of the Danish cohort to those with diagnostic code F84.0 (autistic disorder).

However, despite the lack of reliability with respect to individual diagnoses within the SSC, there were correlations between diagnosis and proband IQ, which also correlates with proband *de novo* rate.[1,34] For example, mean ratio IQ among SSC probands with an Asperger's disorder diagnosis is 110.19 (sd=15.4; n=290). Mean ratio IQ among SSC probands with an autistic disorder diagnosis is 86.2 (sd= 19.8; n=1979). We would therefore expect advanced paternal age to confer somewhat greater risk for phenotypes likely to be diagnosed as autistic disorder as compared to Asperger's disorder.

Therefore, although we determined that the cleanest comparison between our model and the epidemiologic risk for ASD associated with advanced paternal age involved using only cases with the F84.0 diagnostic code, we conducted a supplemental analysis in which we estimated the epidemiologic paternal age effect for individuals with diagnostic codes F84.5 (Asperger's disorder), F84.8 (other pervasive developmental disorders) or F84.9 (PDD-NOS) (see Supplementary Table 8). The hazard ratio representing the paternal age effect for individuals in the Danish population with these diagnostic codes was significantly lower than for F84.0 (1.34 vs. 1.68, two-sample z-test p value = 5.5×10^{-4}).

Although there were more cases in the Danish registry with diagnostic codes of F84.5, F84.8 or F84.9 and fathers who were in their 20s or over 40 at birth (4,970) compared with those with diagnostic code of F84.0 (1,883), the most relevant comparison to the SSC population would be a Danish cohort in which 70% of ASD-spectrum cases are diagnosed with F84.0. To determine the HR associated with advanced paternal age in this hypothetical population, we exponentiate the weighted average of the ln(HRs):

$$e^{0.3*\ln(1.34)+0.7*\ln(1.68)} = 1.57$$

We tested whether the paternal age effect in this hypothetical population is still significantly larger than the paternal age effect seen in the dnSNV model. To do so we needed a standard error for the natural logarithm of this HR which can be found by taking the standard errors for the logarithms of hazard ratios associated with the F84.5/8/9 diagnoses (0.037) and the F84.0 diagnosis (0.054):

$$\mathrm{SE}(\ln(\mathrm{HR}))=\sqrt{0.3^2*0.037^2*0.7^2*0.054^2}=0.039$$

Following the same permutation-based procedure described in the methods we find that the epidemiologic paternal age effect remains significantly larger than the effect predicted by the dnSNV model ($p < 2x10^{-5}$). Thus, our conclusion that the epidemiologic paternal age effect in Denmark cannot be fully accounted for by dnSNVs is robust to the choice we made about how best to capture ASD probands within the Danish registry data.

Supplementary Note 20: Aligning phenotypes for CHD

Because different forms of congenital heart disease may have different genetic architectures and therefore different degrees of risk associated with *de novo* variation, we performed an analysis to investigate the epidemiologic association between paternal age and CHD under conditions where the distribution of CHD types is similar to that seen in the probands of the trio families included in this analysis. Of the 2645 CHD probands, 2531 are from the Pediatric Cardiac Genomics Consortium (PCGC). We first used the Fyler diagnostic codes available for each of 2348 PCGC probands in order to assign probands to one or more broad classes of CHD. Individuals who fit into more than one class were assigned to the more severe class. These classes of CHD are listed in order of severity (with the most severe class at the top) in Table 3. For each Fyler-code-defined class of CHD, we identified corresponding ICD-10 codes, which were then used to query the Danish registry data. Of 2348 PCGC probands, there were 14 whose form of CHD did not fit neatly into any class that could be identified using ICD codes. There were an additional 171 with an atrial septal defect. While probands in the PCGC trio study with atrial septal defects all have severe defects, expert clinical experience

suggests that the vast majority of individuals within the Danish registry with the corresponding ICD-10 code (Q21.1) would have a comparatively benign patent foramen ovale identified at birth or shortly thereafter. Because we are interested in identifying individuals in the Danish registry whose disorders would be eligible for inclusion in the PCGC trio study, we accordingly excluded atrial septal defects (Q21.1) from our query (both for the main analysis and the analysis described here). In addition, we excluded heterotaxy (ICD-10 code of Q20.6) from the Danish analysis as only 11 individuals had the diagnosis and none had a father older than 39 at the time of their birth.

For the 10 remaining classes of congenital heart defects listed in Table 3, we identified each case within the Danish registry who was diagnosed by age 1 and whose father at birth was either in their 20s or older than 39. We also identified the distribution of these 10 classes within the PCGC probands (Table 3). We first sampled this PCGC cases (with replacement) in order to identify phenotypically-corresponding CHD cases identified in the Danish registry whose fathers were either in their 20s or older than 39 at birth (n=1,966; see Table 3). (Some individuals are counted under more than one category in Table 3 so the total number of cases in the Danish registry within this table adds to more than 1,966). We repeated this process 10,000 times to generate a distribution of plausible paternal age distributions in the Danish CHD cases (excluding individuals whose fathers were in their teens or in their 30s). For each iteration, we used the number of non-cases born between 1994-2011 whose fathers were known to be either in their 20s or older than 39 (respective ns = 299,605 and 83,258) in order to calculate an odds ratio associated with advanced paternal age. This procedure gives an

estimated OR of 1.01 (95% CI 0.91 – 1.12), close to the estimate obtained in the main analysis (1.03; 95% CI 0.92-1.14).

Next, we created a distribution of cases weighted according to the frequency with which each class of CHD occurs in the PCGC probands. We similarly sampled 1,966 individuals from this distribution of cases 10,000 times and used the same number of non-cases with paternal ages in their 20s and more than 39 in order to derive an estimate (and 95% CI) of the epidemiologic paternal age effect in a hypothetical Danish population in which the distribution of CHD cases resembles the distribution of CHD cases in the PCGC probands. Following this procedure, we obtained an estimated OR of 1.15 (95% CI 1.03-1.22). We compared the two distributions of ORs to one another in order to derive an empiric p value to test the null hypothesis that there is no difference between 1) the OR reflecting the observed risk for CHD associated with advanced paternal age in the actual Danish population and 2) the same OR in the hypothetical Danish population with the distribution of CHD cases that matches the distribution in the PCGC probands. We did not find statistical evidence that these ORs are different (two-sided p value = .11). The paternal age effect in the hypothetical Danish population was also not statistically different from the observed paternal age effect using our dnSNV model (p = 0.65). The analysis described here suggests that use of a population-based cohort with equal case distribution seen to the PCGC cases would be unlikely to meaningfully impact our analyses.

SUPPLEMENTARY FIGURES:

Supplementary Figure 1

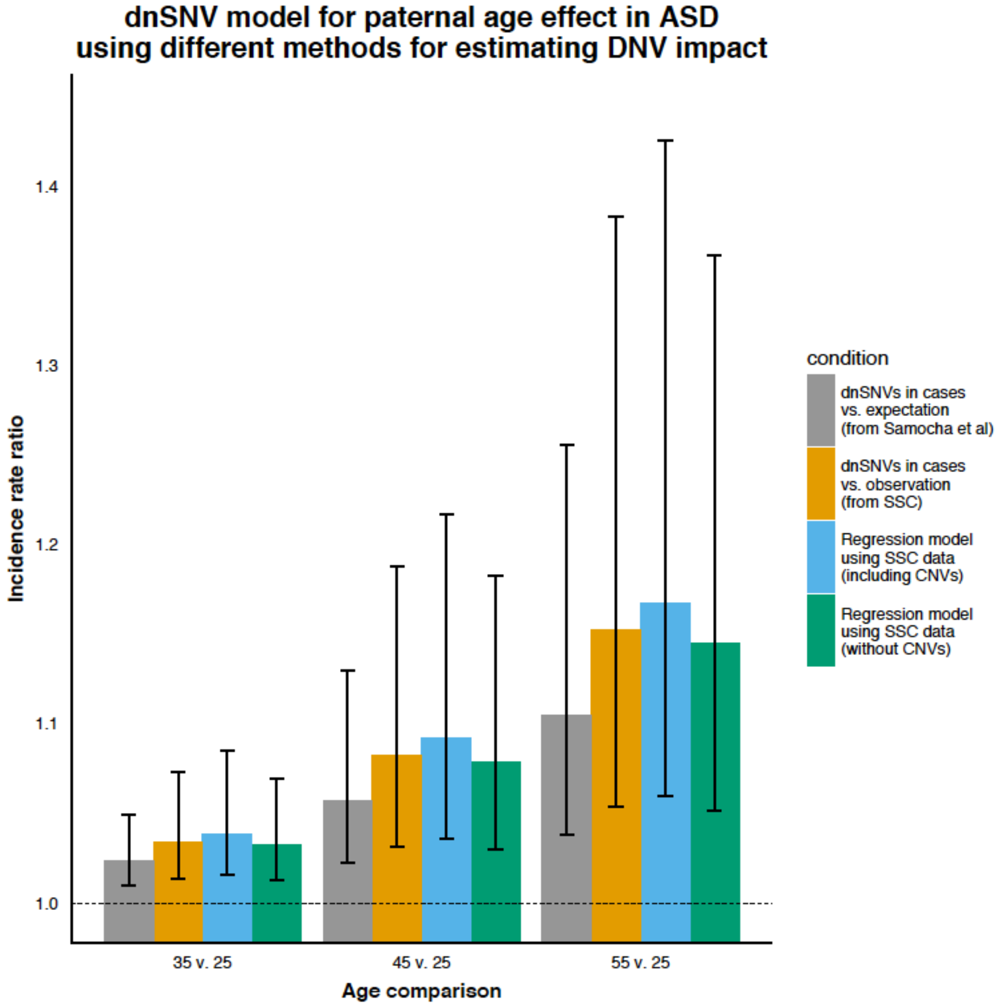

This model for estimating the paternal age effects on autism spectrum disorder (ASD) risk is robust to input variation. This figure presents the expected impact of paternal age on ASD risk for offspring of 35, 45, and 55-year-old fathers compared with the offspring of 25-year-old fathers using four distinct methods for estimating the impact of *de novo* variation. From left to right, colors represent the following methods: (1) Estimating the effect size of *de novo* single nucleotide variants (dnSNVs) by dividing the average number of dnSNVs in ASD probands by the number expected using Samocha et al (2014)'s model; (2) estimating the dnSNV effect size by dividing the number of dnSNVs in ASD probands by the observed number of dnSNVs per unaffected sibling in the Simon Simplex Collection (SSC) quad families (this is the method used to generate the main results); (3) estimating the dnSNV effect size using a Poisson regression model in which number of dnSNVs + number of genotyped copy number variants is regressed on proband/sibling status within the SSC quad families, while controlling for paternal age at birth; the exponentiated coefficient for proband/sibling status is the estimated OR associated with a single *de novo* variant; and (4) a similar Poisson regression model that only takes into account dnSNVs (without CNVs). Error bars reflect 95% confidence intervals.

Supplementary Figure 2

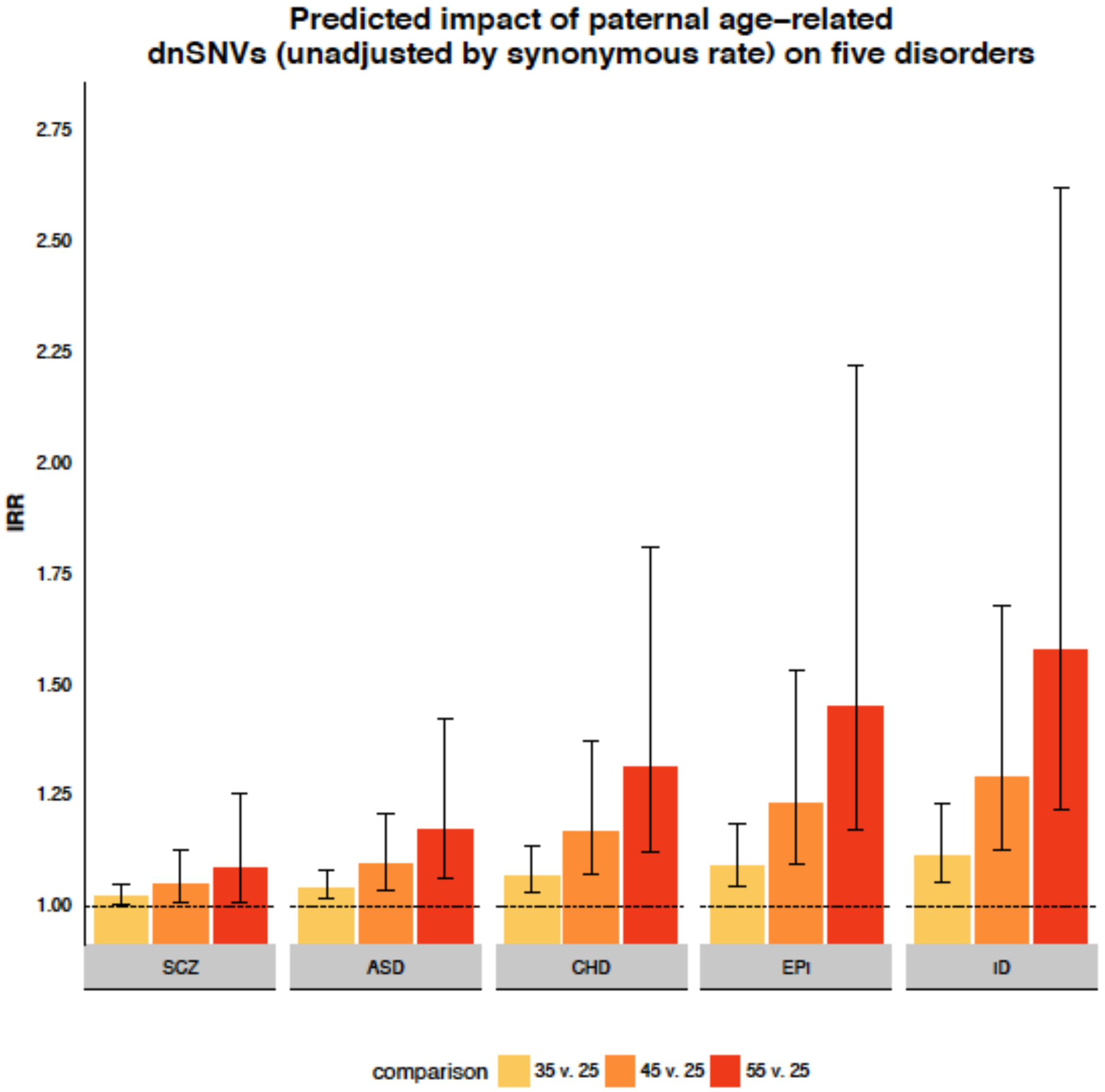

This figure is directly analogous to Figure 2 in the main text. Here, estimated IRRs (incident rate ratios) reflect the influence of nonsynonymous dnSNVs unadjusted by the rate of synonymous dnSNVs (see Supplementary Note 8). SCZ = schizophrenia; ASD = autism spectrum disorder; CHD = congenital heart disease; EPI = epilepsy; ID = intellectual disability. Error bars reflect 95% confidence intervals.

Supplementary Figure 3

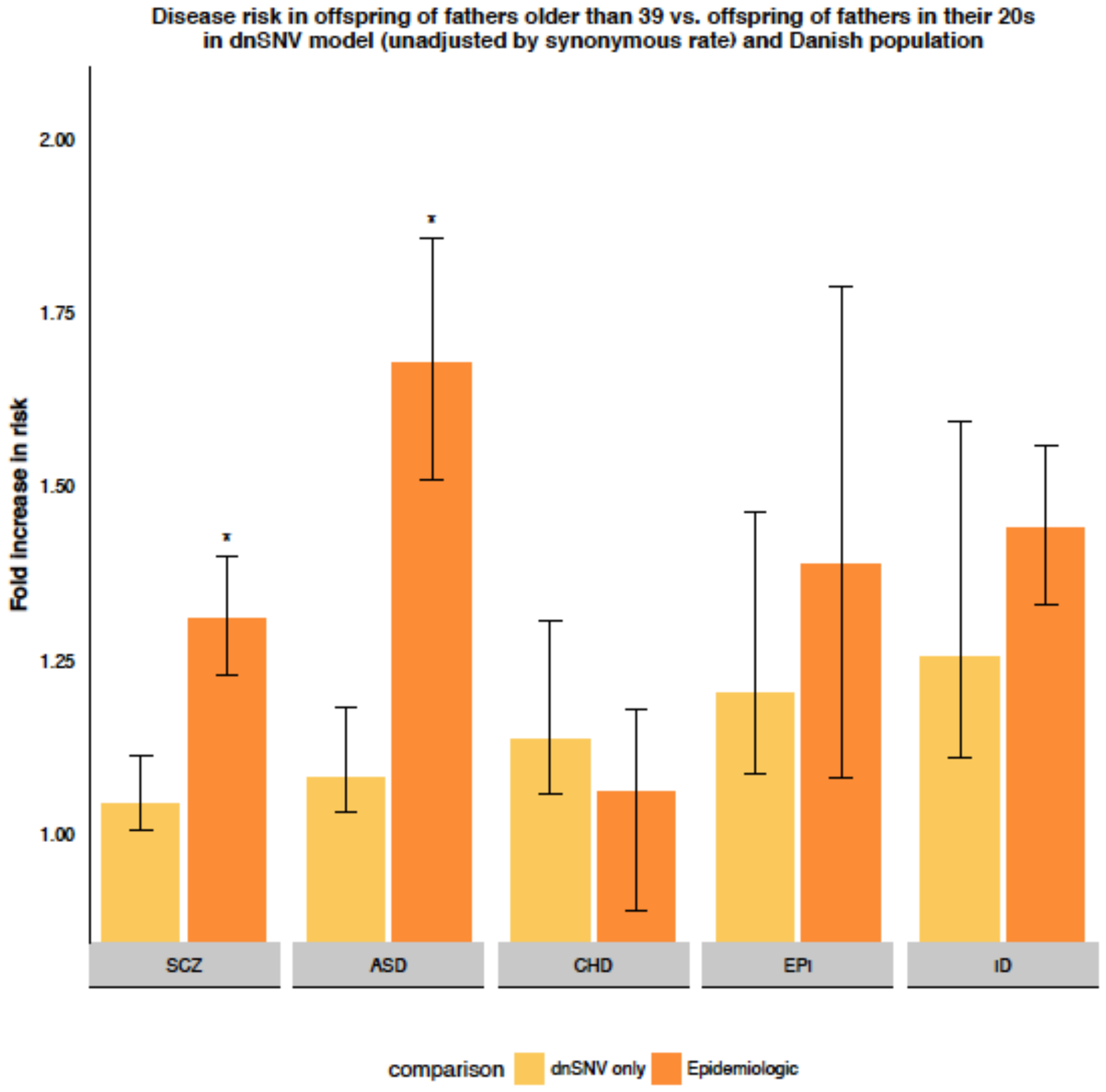

This figure is directly analogous to Figure 3 in the main text. Here, estimates for the fold-increase in risk (IRRs) attributable to paternal-age-related dnSNVs (yellow bars) are not adjusted by the rate of synonymous dnSNVs. SCZ = schizophrenia; ASD = autism spectrum disorder; CHD = congenital heart disease; EPI = epilepsy; ID = intellectual disability. Error bars reflect 95% confidence intervals. * $p < 0.05$ against null hypothesis that epidemiologic association between advanced paternal age and disease risk is equivalent to the dnSNV model's estimate.

Supplementary Figure 4

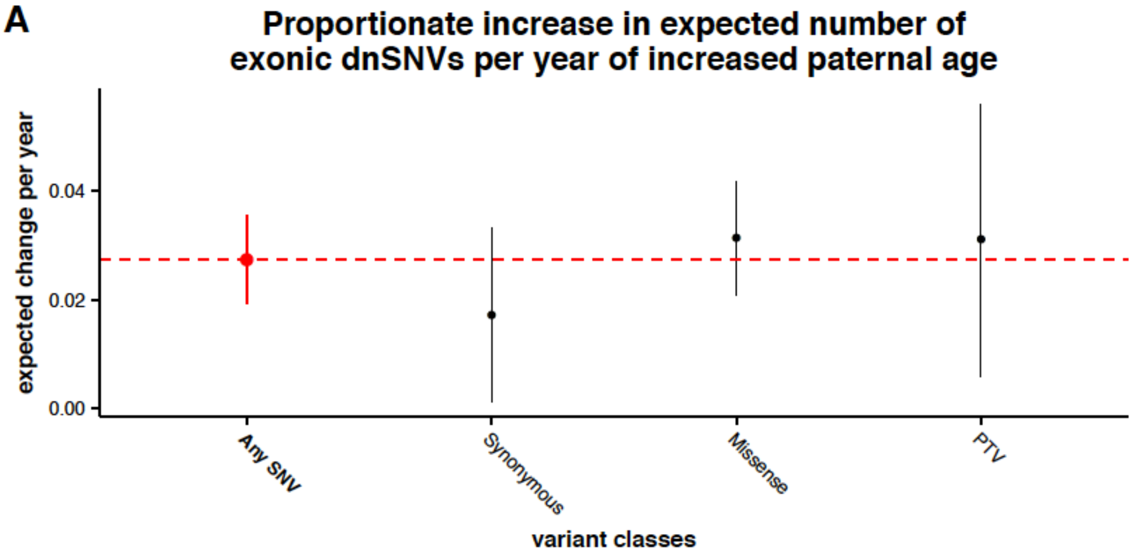

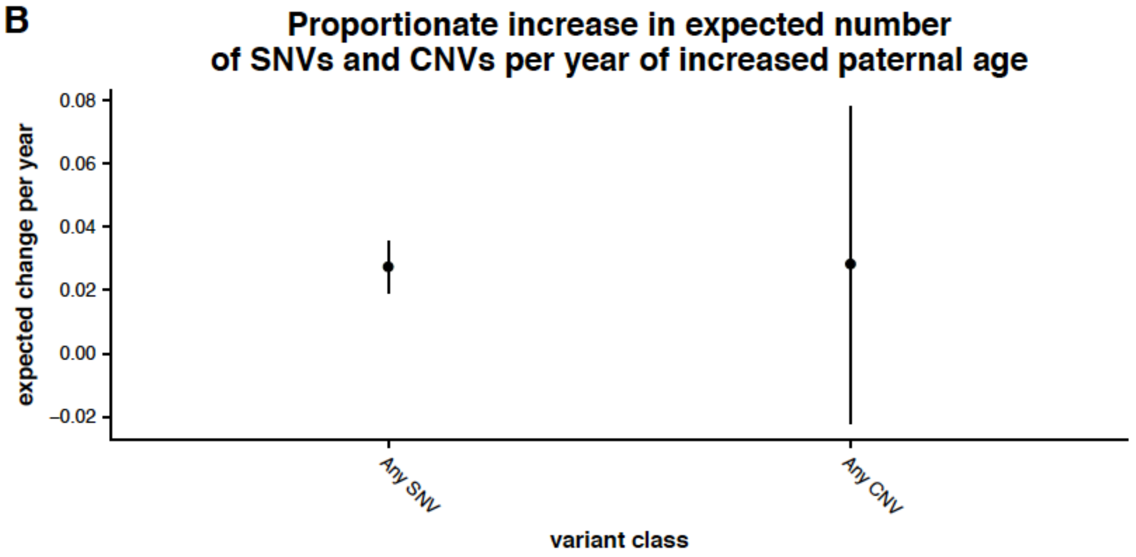

A: The expected number of any exonic dnSNVs (synonymous and nonsynonymous) increased by 2.7% for each year of increased paternal age. This is statistically indistinguishable from the rate calculated directly for nonsynonymous dnSNVs (0.031) (z-test p-value = 0.40). There is likewise no significant difference in rate between synonymous and non-synonymous dnSNVs (z-test p-value = 0.14). The class of exonic variant – synonymous vs. missense vs. protein-truncating (PTV) – does not substantively impact this rate. Error bars represent 95% confidence intervals. B: At current sample sizes, the rate of change in *de novo* copy number variants (dnCNVs) with respect to paternal age is statistically indistinguishable from both zero and the rate of change in *de novo* single nucleotide variants (dnSNVs). Bars represent 95% confidence intervals.

Supplementary Figure 5

A) Mean number of dnSNVs by paternal age decile vs proportional model

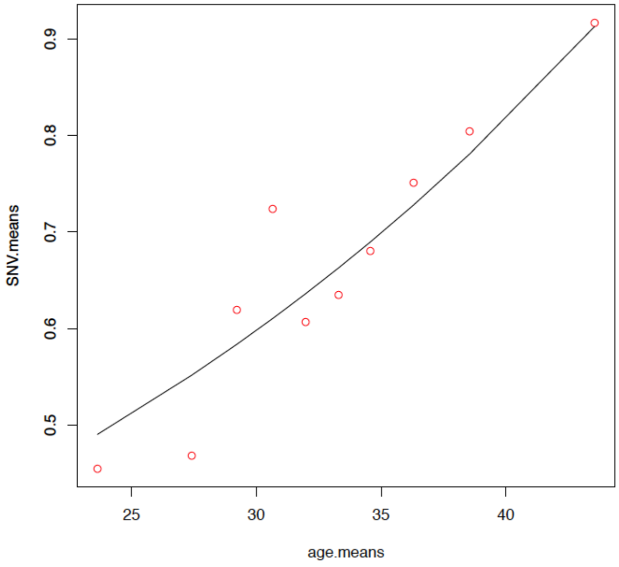

B) Mean number of dnSNVs by paternal age decile vs linear model

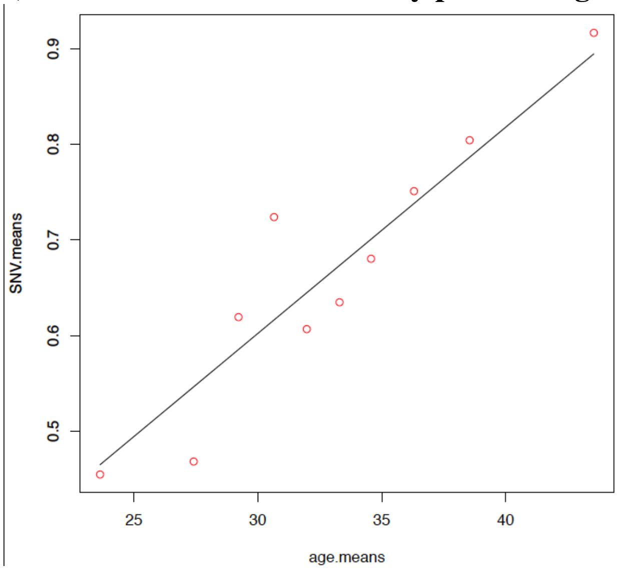

We divided all 1827 SSC siblings into deciles defined by paternal ages. For each decile we plotted the mean paternal age against the mean number of dnSNVs. We compared this representation of the data to a model of the relationship derived using Poisson regression (5a) and linear regression (5b).

Supplementary Tables

Supplementary Table 1: Incidence rate ratios reflecting dnSNV-mediated increased risk for 5 disorders in offspring of older fathers compared with offspring of 25 year old fathers (unadjusted for rate of synonymous dnSNVs)

older age	ASD	SCZ	ID	CHD	EPI
**30**	1.02 (1.01-1.04)	1.01 (1.00-1.02)	1.05 (1.02-1.10)	1.03 (1.01-1.06)	1.04 (1.02-1.08)
**35**	1.04 (1.02-1.08)	1.02 (1.00-1.05)	1.11 (1.05-1.23)	1.07 (1.03-1.13)	1.09 (1.04-1.19)
**40**	1.06 (1.03-1.14)	1.03 (1.00-1.08)	1.19 (1.09-1.41)	1.11 (1.05-1.23)	1.15 (1.07-1.33)
**45**	1.09 (1.04-1.21)	1.05 (1.01-1.13)	1.29 (1.13-1.68)	1.17 (1.07-1.37)	1.23 (1.10-1.53)
**50**	1.13 (1.05-1.31)	1.07 (1.01-1.19)	1.42 (1.17-2.08)	1.23 (1.09-1.55)	1.33 (1.13-1.81)
**55**	1.17 (1.06-1.42)	1.09 (1.01-1.26)	1.58 (1.22-2.62)	1.32 (1.12-1.81)	1.45 (1.17-2.22)
**60**	1.22 (1.08-1.61)	1.11 (1.01-1.35)	1.79 (1.28-3.63)	1.42 (1.15-2.16)	1.61 (1.22-2.83)
**65**	1.29 (1.10-1.84)	1.14 (1.02-1.46)	2.08 (1.35-5.18)	1.55 (1.19-2.74)	1.81 (1.27-3.87)
**70**	1.37 (1.12-2.14)	1.18 (1.02-1.62)	2.47 (1.42-8.42)	1.72 (1.23-3.69)	2.08 (1.33-5.66)
**75**	1.47 (1.14-2.61)	1.22 (1.03-1.85)	3.02 (1.53-15.40)	1.94 (1.28-5.07)	2.46 (1.41-9.63)

Supplementary Table 1 Note: This table is analogous to Supplementary Table 2. It presents results where we do not adjust for rate of synonymous variants, as described in Supplementary Note 8.

Supplementary Table 2: Comparing dnSNV model to Danish epidemiologic data (unadjusted for rate of synonymous variation)

Disorder	paternal age comparison	de novo only effect (95% CI)	Epidemiologic association (95% CI)	p value
SCZ	26.2 vs. 44.1	1.04 (1.00 - 1.11)	1.31(1.23-1.40)	$1.4x10^{-4}$
ASD	26.2 vs. 44.1	1.08 (1.03 - 1.18)	1.68(1.51-1.86)	$<2x10^{-05}$
CHD	27.1 vs. 43.7	1.14 (1.06 - 1.31)	1.06(0.89-1.18)	0.29
EP	26.2 vs. 44.1	1.20 (1.09 - 1.47)	1.39 (1.08-1.79)	0.37
ID	26.2 vs. 44.1	1.26 (1.11 - 1.60)	1.44(1.33-1.61)	0.24

Supplementary Table 2 Note: This table is analogous to Supplementary Table 3. It presents results where we do not adjust for rate of synonymous variants, as described in Supplementary Note 8

Supplementary Table 3: The reported rate of increase in dnSNVs associated with each year of increased paternal age in whole-genome trio sequencing studies

Reference	# of Trios	Description of data as published	Transformation performed	% increase in SNVs per year of increased paternal and maternal ages
24	78	"When an exponential model is fitted…the number of paternal and maternal mutations combined is estimated to increase by 3.23% per year"	none	3.2
25	258	"we estimated that an offspring born to a 20-year-old father would receive on average 9.63 genic and 22.68 intergenic mutations, whereas an offspring of a 40-year-old father would receive on average 19.06 genic and 35.24 intergenic mutations."	assuming that dnSNVs are Poisson distributed leads to two simultaneous equations that can be solved for beta: (1) exp(i + 20*beta) = 9.63 + 22.68; (2) exp(i + 40*beta) = 19.06 + 35.24	2.6

Reference	# of Trios	Description of data as published	Transformation performed	% increase in SNVs per year of increased paternal and maternal ages
35	816	The authors report an average of 45 dnSNVs per proband, an increase of 0.91 per year of increased paternal age, and an increase of 0.24 per year of increased maternal age	(0.91 + 0.24)/45 = 0.026	2.6
36	13	The authors report an estimated 76.9 mutations per individual with an increase of 2.87 mutations per year of increased paternal age (no adjustment for maternal age)	2.87/76.9 = 0.037	3.7
37	10	Figure 1 graphically illustrates paternal age (x-axis) and number of dnSNVs (y axis) for each of 10 probands	We used built-in functionality on Mac OS to estimate the location of each point in this graph and then ran a Poisson model regressing number of dnSNVs on paternal age	3.4

Supplementary Table 3 Note: We reviewed existing whole-genome sequencing studies of trio families that report an association between parental age and number of *de novo* single nucleotide variants (dnSNVs). In most cases these studies did not directly describe their results in terms of percent increase in dnSNVs per year of parental age. Therefore, to make these results comparable to ours, we describe the transformations we performed on the data in order to arrive at the % increase in SNVs per year of increased

parental age. The reported rate of increase in dnSNVs in whole-genome trio studies ranged from 2.6% to 3.7%, suggesting that the rate we found for nonsynonymous, exonic dnSNVs (3.1%) is consistent with those previously published using whole-genome data.

Supplementary Table 4: Incidence rate ratios reflecting dnSNV-mediated increased risk for 5 disorders in offspring of older fathers compared with offspring of 25 year old fathers

older age	ASD	SCZ	ID	CHD	EPI
**30**	1.02 (1.00-1.04)	1.02 (1.00-1.04)	1.04 (1.02-1.07)	1.02 (1.01-1.05)	1.03 (1.01-1.07)
**35**	1.04 (1.01-1.09)	1.04 (1.00-1.10)	1.08 (1.03-1.17)	1.05 (1.02-1.12)	1.08 (1.03-1.17)
**40**	1.06 (1.01-1.15)	1.06 (1.00-1.17)	1.13 (1.06-1.29)	1.09 (1.03-1.20)	1.13 (1.05-1.30)
**45**	1.09 (1.02-1.23)	1.09 (1.01-1.26)	1.20 (1.08-1.47)	1.13 (1.04-1.31)	1.19 (1.07-1.47)
**50**	1.12 (1.02-1.33)	1.13 (1.01-1.39)	1.28 (1.11-1.71)	1.18 (1.06-1.46)	1.27 (1.09-1.70)
**55**	1.16 (1.03-1.46)	1.17 (1.01-1.54)	1.38 (1.14-2.05)	1.24 (1.08-1.65)	1.36 (1.12-2.04)
**60**	1.21 (1.04-1.66)	1.22 (1.01-1.77)	1.51 (1.18-2.59)	1.32 (1.10-1.95)	1.48 (1.15-2.55)
**65**	1.27 (1.05-1.92)	1.28 (1.02-2.07)	1.68 (1.22-3.37)	1.41 (1.12-2.36)	1.64 (1.19-3.32)
**70**	1.34 (1.06-2.24)	1.36 (1.02-2.52)	1.9 (1.26-4.80)	1.53 (1.14-3.05)	1.85 (1.23-4.63)
**75**	1.43 (1.07-2.74)	1.46 (1.03-3.24)	2.2 (1.33-7.44)	1.69 (1.18-3.91)	2.12 (1.28-7.39)

Supplementary Table 4 Note: Detailed results of dnSNV model across disorders and paternal ages. Numbers in each box reflect incidence rate ratios representing the increased risk due to dnSNVs in offspring of fathers with ages in the table’s first column compared with offspring of 25-year-old fathers. Parentheticals reflect 95% confidence intervals.

Supplementary Table 5: Comparing dnSNV model to Danish epidemiologic data

Disorder	paternal age comparison	de novo only effect (95% CI)	Epidemiologic association (95% CI)	p value	P value assuming dnSNVs do not accumulate until age 13
SCZ	26.2 vs. 44.1	1.08 (1.00-1.23)	1.31 (1.23-1.40)	0.02	0.006
ASD	26.2 vs. 44.1	1.08(1.20-1.02)	1.68 (1.51-1.86)	$2x10^{-5}$	$<2x10^{-5}$
CHD	27.1 vs. 43.7	1.11(1.04-1.26)	1.06 (0.89-1.18)	0.50	0.48
EP	26.2 vs. 44.1	1.17(1.06-1.40)	1.39 (1.08-1.79)	0.27	0.23
ID	26.2 vs. 44.1	1.17(1.07-1.41)	1.44 (1.33-1.61)	0.05	0.02

Supplementary Table 5 Note: For each disorder we note the mean paternal ages of the two groups of Danish individuals compared. Using these two ages, we run the dnSNV model for each disorder and describe the expected increase in risk for offspring of older fathers as an incidence rate ratio with a 95% confidence interval. We then note the epidemiologic association in Denmark between the two groups with 95% confidence intervals. These associations are expressed as a hazard ratio for schizophrenia (SCZ), autism (ASD), epilepsy (EPI) and intellectual disability (ID) and as an odds ratio for congenital heart disease (CHD). P-values are the empiric 2-sided p values for the null hypothesis that the increased risk in offspring of older fathers accounted for by the dnSNV model is equivalent to the epidemiologic association observed in Denmark. The method for generating these p values are described in detail in Methods. Finally, we report p-values that would result from making the anti-conservative choice to assume that dnSNVs do not start accumulating until age 13 (see Supplementary Note 9).

Supplementary Table 6: De novo synonymous variation across cohorts

Cohort	N trios	synonymous dnSNVs per person	OR (p)
Intellectual disability	5264	0.29	1.18 (0.002)
Neurodevelopmental disorders with epilepsy	1942	0.18	1.08 (0.33)
Congenital heart disease	2645	0.27	1.08 (0.22)
Autism spectrum disorders	2508	0.25	1.01 (0.83)
Schizophrenia	1077	0.22	0.91 (0.26)
Control	1902	0.25	-
Control (epilepsy)	1911	0.17	-

Supplementary Table 6 Note: This table is analogous to Table 1 in the main text. Here we show the rates of synonymous variants for each disorder. OR is the rate of synonymous dnSNVs per person in affected probands divided by the same rate in Simons Simplex Collection control siblings. The associated p values were generated using a Poisson exact test. Note that the only disorder phenotype for which there was a statistically significant difference in synonymous rate compared with the SSC siblings was ID.

Supplementary Table 7: **Details of Danish epidemiologic analyses**

Disorder	mean paternal age (younger age bin)	mean paternal age (older age bin)	cases (younger age bin)	person-time in years / number of non-cases (younger age bin)	cases (older age bin)
**SCZ**	26.2	44.1	6,982	21,453,197	1,034
**ASD**	26.2	44.1	1,423	24,666,267	460
**EP**	26.2	44.1	307	6,210,827	77
**ID**	26.2	44.1	3,380	24,623,537	749
**CHD**	27.1	43.7	1,529	299,605	437

Disorder	person-time in years / number of non-cases (older age bin)	Crude HR/OR (95% CI)	SE(log(HR/OR))	HR/OR adjusted for maternal age (95% CI)	HR/OR adjusted for calendar time (95% CI)
**SCZ**	2,816,928	1.31 (1.23-1.40)	0.033	1.40 (1.29-1.51)	1.31 (1.23-1.40)
**ASD**	3,534,847	1.68 (1.51-1.86)	0.054	1.52 (1.34-1.71)	1.45 (1.30-1.61)
**EP**	1,148,615	1.39 (1.08-1.79)	0.128	1.52 (1.14-2.03)	1.36 (1.06-1.74)
**ID**	3,527,115	1.44 (1.33-1.56)	0.041	1.46 (1.33-1.61)	1.40 (1.29-1.52)
**CHD**	83,258	1.03 (0.92-1.14)	0.052	1.00 (0.89-1.13)	-

Supplementary Table 7 note: For each disorder, the mean paternal ages refer to those at risk in our analyses using the Danish registries whose fathers were in their 20s (younger) and over age 39 (older) at the time of their births. For SCZ, ASD, EPI and ID, the cohort for determining the mean age for each age bin is all those in the Danish registries with available data on paternal age born between 1955-2012. For CHD, the cohort for determining mean ages are those born between 1994-2012. For each disorder we report total number of cases ascertained through the registries. We also

report total person-time at risk in each age bin for SCZ, ASD, EPI and ID and total number of individuals at risk in each age bin for CHD. We report the crude hazard ratios (odds ratio for CHD) with standard error of the natural logarithm of these estimates, as well as the results of adjusting for maternal age, or calendar year.

Supplementary Table 8: Danish epidemiologic analysis for alternative ASD diagnostic codes (F84.5, F84.8, F84.9)

mean paternal age (younger age bin)	mean paternal age (older age bin)	cases (younger age bin)	person-time in years / number of non-cases (younger age bin)	cases (older age bin)
26.2	44.1	4,063	24,648,865	907

person-time in years / number of non-cases (older age bin)	Crude HR/OR (95% CI)	SE(log(HR/OR))	HR/OR adjusted for maternal age (95% CI)	HR/OR adjusted for calendar time (95% CI)
3,532,366	1.34 (1.24-1.44)	0.037	1.28 (1.18-1.39)	1.19 (1.11-1.28)

Supplementary Table 8 note: This table follows the same format as Supplementary Table 7. It shows the results of an alternative approach to capturing the ASD phenotype in the Danish registry data that is further described in Supplementary Note 19.

Supplementary References

1. Iossifov, I. *et al.* The contribution of de novo coding mutations to autism spectrum disorder. *Nature* **515**, 216-21 (2014).
2. Girard, S.L. *et al.* Increased exonic de novo mutation rate in individuals with schizophrenia. *Nat Genet* **43**, 860-3 (2011).
3. Xu, B. *et al.* Exome sequencing supports a de novo mutational paradigm for schizophrenia. *Nat Genet* **43**, 864-8 (2011).
4. Xu, B. *et al.* De novo gene mutations highlight patterns of genetic and neural complexity in schizophrenia. *Nat Genet* **44**, 1365-9 (2012).
5. Gulsuner, S. *et al.* Spatial and temporal mapping of de novo mutations in schizophrenia to a fetal prefrontal cortical network. *Cell* **154**, 518-29 (2013).
6. De Rubeis, S. *et al.* Synaptic, transcriptional and chromatin genes disrupted in autism. *Nature* **515**, 209-215 (2014).
7. Takata, A. *et al.* Loss-of-function variants in schizophrenia risk and SETD1A as a candidate susceptibility gene. *Neuron* **82**, 773-80 (2014).
8. McCarthy, S.E. *et al.* De novo mutations in schizophrenia implicate chromatin remodeling and support a genetic overlap with autism and intellectual disability. *Mol Psychiatry* **19**, 652-8 (2014).
9. Guipponi, M. *et al.* Exome sequencing in 53 sporadic cases of schizophrenia identifies 18 putative candidate genes. *PLoS One* **9**, e112745 (2014).
10. de Ligt, J. *et al.* Diagnostic exome sequencing in persons with severe intellectual disability. *N Engl J Med* **367**, 1921-9 (2012).
11. Rauch, A. *et al.* Range of genetic mutations associated with severe non-syndromic sporadic intellectual disability: an exome sequencing study. *Lancet* **380**, 1674-82 (2012).
12. Lelieveld, S.H. *et al.* Meta-analysis of 2,104 trios provides support for 10 new genes for intellectual disability. *Nat Neurosci* **19**, 1194-6 (2016).
13. Deciphering Developmental Disorders, S. Large-scale discovery of novel genetic causes of developmental disorders. *Nature* **519**, 223-8 (2015).
14. Deciphering Developmental Disorders, S. Prevalence and architecture of de novo mutations in developmental disorders. *Nature* **542**, 433-438 (2017).
15. Heyne, H.O. *et al.* The Spectrum Of De Novo Variants In Neurodevelopmental Disorders With Epilepsy. *bioRxiv* (2017).
16. Homsy, J. *et al.* De novo mutations in congenital heart disease with neurodevelopmental and other congenital anomalies. *Science* **350**, 1262-6 (2015).
17. Lim, E.T. *et al.* Rates, distribution and implications of postzygotic mosaic mutations in autism spectrum disorder. *Nat Neurosci* **20**, 1217-1224 (2017).
18. Samocha, K.E. *et al.* A framework for the interpretation of de novo mutation in human disease. *Nat Genet* **46**, 944-50 (2014).
19. Sanders, S.J. *et al.* Insights into Autism Spectrum Disorder Genomic Architecture and Biology from 71 Risk Loci. *Neuron* **87**, 1215-33 (2015).
20. Goriely, A. & Wilkie, A.O. Paternal age effect mutations and selfish spermatogonial selection: causes and consequences for human disease. *Am J Hum Genet* **90**, 175-200 (2012).

21. Gratten, J. *et al.* Risk of psychiatric illness from advanced paternal age is not predominantly from de novo mutations. *Nat Genet* **48**, 718-24 (2016).
22. Fombonne, E. The prevalence of autism. *Jama* **289**, 87-89 (2003).
23. prevention, C.f.d.c.a. Autism spectrum disorders (ASDs): data & statistics. (Centers for Disease Control and Prevention, 2016).
24. Kong, A. *et al.* Rate of de novo mutations and the importance of father's age to disease risk. *Nature* **488**, 471-5 (2012).
25. Genome of the Netherlands, C. Whole-genome sequence variation, population structure and demographic history of the Dutch population. *Nat Genet* **46**, 818-25 (2014).
26. Wong, W.S. *et al.* New observations on maternal age effect on germline de novo mutations. *Nat Commun* **7**, 10486 (2016).
27. eurostat. Marriage indicators: Mean age at first marriage - males. 5/30/2017 edn (2017).
28. eurostat. Marriage indicators: Mean age at first marriage - females. 5/30/2017 edn (2017).
29. Samocha, K.E. *et al.* Regional missense constraint improves variant deleteriousness prediction. *bioRxiv* (2017).
30. Fromer, M. *et al.* De novo mutations in schizophrenia implicate synaptic networks. *Nature* **506**, 179-84 (2014).
31. Jin, S.C. *et al.* Contribution of rare inherited and de novo variants in 2,871 congenital heart disease probands. *Nat Genet* **49**, 1593-1601 (2017).
32. Lord, C. *et al.* A multisite study of the clinical diagnosis of different autism spectrum disorders. *Arch Gen Psychiatry* **69**, 306-13 (2012).
33. Association, A.P. *Diagnostic and statistical manual of mental disorders (DSM-5®)*, (American Psychiatric Pub, 2013).
34. Robinson, E.B. *et al.* Autism spectrum disorder severity reflects the average contribution of de novo and familial influences. *Proc Natl Acad Sci U S A* **111**, 15161-5 (2014).
35. Goldmann J.M. *et al*. Parent-of-origin-specific signatures of de novo mutations. *Nat Genet* **48,** 935-9 (2016).
36. Rahbari R. *et al.* Timing, rates and spectra of human germline mutation. N*at Genet* **48,** 126-33 (2016).
37. Michaelson J.J. *et al*. Whole-genome sequencing in autism identifies hot spots for de novo germline mutation. Cell **151,** 1431-42 (2012).
